# Motor Effort Alters Changes of Mind in Sensorimotor Decision Making

**DOI:** 10.1371/journal.pone.0092681

**Published:** 2014-03-20

**Authors:** Diana Burk, James N. Ingram, David W. Franklin, Michael N. Shadlen, Daniel M. Wolpert

**Affiliations:** 1 Computational and Biological Learning Laboratory, Department of Engineering, University of Cambridge, Cambridge, United Kingdom; 2 Howard Hughes Medical Institute, Janelia Farm Research Campus, Ashburn, Virginia, United States of America; 3 Intramural Research Program, National Institute on Drug Abuse, Baltimore, Maryland, United States of America; 4 Howard Hughes Medical Institute, Kavli Institute and Department of Neuroscience, Zuckerman Mind Brain Behavior Institute, Columbia University, New York, New York, United States of America; Max Planck Institute for Human Cognitive and Brain Sciences, Germany

## Abstract

After committing to an action, a decision-maker can change their mind to revise the action. Such changes of mind can even occur when the stream of information that led to the action is curtailed at movement onset. This is explained by the time delays in sensory processing and motor planning which lead to a component at the end of the sensory stream that can only be processed after initiation. Such post-initiation processing can explain the pattern of changes of mind by asserting an accumulation of additional evidence to a criterion level, termed change-of-mind bound. Here we test the hypothesis that physical effort associated with the movement required to change one's mind affects the level of the change-of-mind bound and the time for post-initiation deliberation. We varied the effort required to change from one choice target to another in a reaching movement by varying the geometry of the choice targets or by applying a force field between the targets. We show that there is a reduction in the frequency of change of mind when the separation of the choice targets would require a larger excursion of the hand from the initial to the opposite choice. The reduction is best explained by an increase in the evidence required for changes of mind and a reduced time period of integration after the initial decision. Thus the criteria to revise an initial choice is sensitive to energetic costs.

## Introduction

A decision is a commitment to a proposition or plan amongst alternatives. Many decisions benefit from deliberation and an accumulation of evidence over time, for example, to improve the accuracy when based on noisy or unreliable evidence [1]–[5]. To deploy this strategy, the brain must also establish a rule for terminating deliberation and committing to a choice. A class of models, termed bounded drift-diffusion or random walk, has been applied to a wide range of decisions to explain both the speed and accuracy of choices made by humans and animals [1]–[11]. The essential feature is an accumulation of signal plus noise until cessation of the evidence stream or until the accumulation reaches a bound.

**Figure 1:**
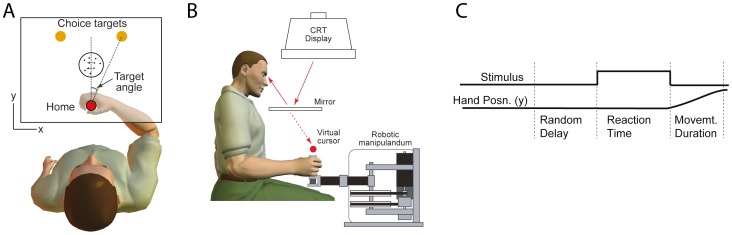
Experimental set-up. A: Schematic of the visual display (rectangle). A trial starts when the subject's hand is in the home position. After a random delay, the random dot kinematogram become visible and the subject views the moving dot stimulus for as long as they need (up to 2 s). Subjects indicate the direction of dot motion by moving to the leftward or rightward choice target. As soon as the subject moves from the home position, the motion stimulus vanished. The trial ended when the subject reached one of the two choice targets. B: Subjects held the handle of a robotic interface and moved to either a leftward or a rightward circular target depending on the perceived motion direction of a central random-dot display. A mirror system prevented subjects from seeing their arm. C: The time course of events that make up a trial.

**Figure 2:**
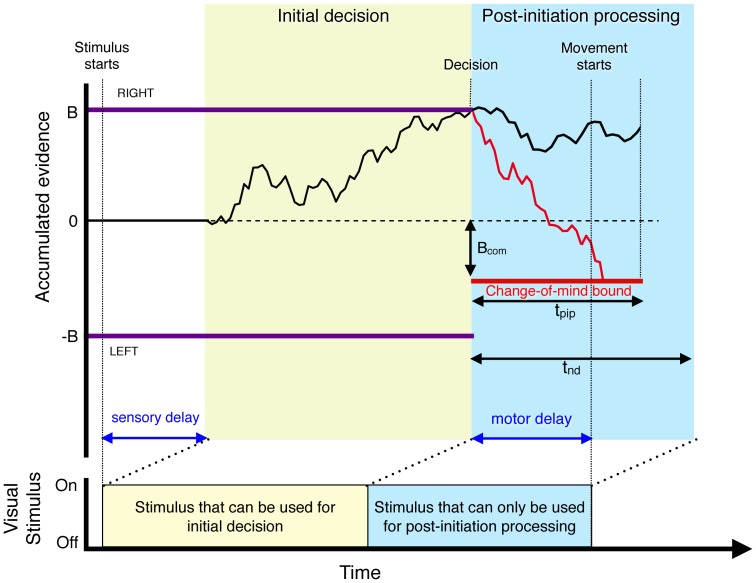
Schematic of a model that explains the pattern of changes of mind [3]. Information flow diagram showing visual stimulus and events leading to a decision and a possible change of mind. The example illustrates a leftward motion stimulus that gives rise to an initial incorrect rightward choice. The visual stimulus gives rise to a decision variable that reflects accumulated evidence (black trace) that is the integral of noisy evidence. This governs the initial choice and decision time. The initial decision is complete when a ‘Right’ or ‘Left’ bound is crossed. There is a sensory delay from motion onset to the beginning of the accumulation and a motor delay from the initial decision to movement initiation. These delays together are termed non-decision time (t_nd_). After the initial decision, further accumulation takes place on the evidence still in the processing pipeline; if the accumulated evidence reaches the opposite change-of-mind bound (B_com_) within a temporal deadline (t_pip_) then the decision is reversed (red). Failure to reach the change-of-mind bound (e.g. black trace) leads to no change of mind. Note that due to the time delays, only the yellow part of the visual stimulus can influence the initial choice and the blue portion can only be processed after the initial choice.

This framework applies particularly well to some perceptual decisions, which must be based on a stream of noisy, independent samples of evidence. For example, when asked to decide the direction of dynamic random dot motion (Fig. 1A), humans and monkeys exhibit choice and reaction time (RT) functions that are explained by the signal-to-noise associated with motion strength and pair of symmetrically placed termination bounds [2], [6], [12] (Fig. 2).

The drift-diffusion framework has been extended recently to explain a curious phenomenon, termed change of mind (CoM), which occurs when a subject indicates a decision about motion direction by reaching towards a choice target (Fig. 1). On a fraction of trials, the subject changes the trajectory of the reach to indicate the other choice [3]. This occurs despite the fact that the sensory stimulus is extinguished the moment the hand begins to move from the starting position (Fig. 1, home). Consideration of the sensory and motor delays leads to an understanding of these change-of-mind trials. These delays, which are on the order or 400 ms, imply that there is a period of information at the end of the sensory stream that cannot affect the initial choice but could still be processed after initiation. A simple extension of the drift diffusion model was able to explain the patterns of changes of mind. The key idea is that having accumulated evidence to one choice bound — thereby triggering the initial choice — a new change-of-mind bound is generated (Fig. 2). If the total accumulated information from the initial decision and the extra processing time crosses this bound (red line) then the subject revises the decision and reaches to the other target. Fits of the model showed that the change of mind bound did not require as much information as for the initial decision and also that not all the information in the processing pipeline was used, that is there was a limited time for which new information was processed.

There is something unsatisfying in this explanation. Consider the optimal strategy for revising an initial decision so as to make the final choice as accurate as possible. This would require accumulating all the available information in the processing pipeline (i.e., the period that includes the sensory and motor delays) and changing one's mind if and only if the final accumulated evidence had a sign opposite to that of the initial choice. However, in our task subjects required more evidence than this to change their mind, and they often terminated further processing by using only a portion of the available evidence. This is captured by a change-of-mind bound on the other side of zero from the bound that was initially crossed and a limited period of time which serves to terminate processing in the post-initiation phase. One possible reason for this apparent sub-optimal behavior is that it requires motor effort to alter the reach to the other choice target. Moreover the amount of motor effort increases as the reach progresses, since the two choice targets are separated in space. Therefore, there may be a trade-off between motor effort and the willingness to change one's mind. To test this hypothesis we manipulated the movement effort that would be required to change the movement from one choice target to the other. We examined how this manipulation affected the frequency of changes of mind and the mechanism relating the drift-diffusion model to motor effort.

## Methods

The Cambridge Psychology Research Ethics Committee approved the procedures in the study. Participants provided written informed consent prior to participating in this study. Four naïve right-handed subjects (1 female, 3 male age 22–30) participated in the study. One additional subject was excluded during initial perceptual training, because this subject performed close to chance.

Subjects were seated and used their right hand to hold the handle of a vBOT manipulandum that was free to move in the horizontal plane (Fig. 1A & B) [13]. The manipulandum allowed the recording of position of the handle and the application of end-point forces. Subjects were prevented from seeing their arm by a mirror that was used to overlay virtual images of a video display (updated at 75 Hz) onto the plane of the movement. A headrest ensured a viewing distance of 40 cm. The hand position was displayed as a small circle (0.5 cm radius).

The timeline of a trial is shown in Fig. 1C). A trial began when the subject's hand was in the home position (1 cm radius). To ensure subjects started from a consistent starting position, when the hand crossed the edge of the home position, a small force guided the hand to the center. After a random delay, sampled from a truncated exponential distribution (range, 0.7–1.0 s; mean, 0.82 s), a dynamic random-dot stimulus appeared at the center of the screen within a circular aperture subtending 5° of visual angle. The motion stimulus is described in detail in previous studies [14]. In each trial, the direction of motion was randomly chosen to be leftward or rightward and had a stimulus density of 15.6 dots deg^−2^ s^−1^. Dots were displayed for one video frame and then either replaced at a random position or displaced to the left or right three video frames (40 ms) later. This displacement would produce a speed of 7.1° s^−1^. Thus the positions of the dots in frame four, say, could only be correlated with dots in frames one and/or seven but not with dots in frames two, three, five and six. The probability that each dot would be displaced as opposed to randomly replaced, termed the coherence, determined the task difficulty and was selected randomly from the set (0, 0.032, 0.064, 0.128, 0.256 and 0.512).

The subjects were instructed to judge the direction of the moving random dots and to reach to a corresponding choice target when ready (one on the left and one on the right; radius, 1.5 cm; 20 cm from the starting position; Fig. 1a). Critically, when the movement was initiated—that is, the hand crossed the boundary of the home-position—the random-dot stimulus was extinguished. Subjects were given feedback if they did not reach the target with a movement duration of 500±100 ms. The trial ended when the subject reached one of the targets. Subjects were provided with visual and auditory feedback of whether they had made the correct choice (and on a random half of the 0 coherence trials). Subjects were instructed to fixate at the centre of the dot aperture (a small fixation cross was presented at the centre when the dots were not in motion to assist this) —the targets were large enough that they could be easily reached using peripheral vision.

Subjects performed 4 different conditions. For three of the conditions, the targets were placed at the three angular positions of 15, 30, and 45° (angle definition shown in Fig. 1A). In a fourth condition, the targets were set at 15° and a resistive, velocity-dependent force-field with a strength of 0.5 N cm^−1^ s (for Subject 2) or 0.9 N cm^−1^ s (for all other subjects) was activated if the hand crossed into the area between the two targets.

To require subjects to make an initial commitment to a choice we simulated a visual and haptic wedge with its tip at the edge of the home position. The sides of the wedge were 0.5 cm long and the wedge angle was 75% of the target angle. If the subject hit the wedge, the current trial was aborted. A new trial was randomly generated and the aborted trial was scheduled to be performed again later in the session.

A session consisted of a four blocks of 334 trials, one for each condition. The order of the blocks (i.e. conditions) was randomized for each session. Each block included roughly an equal number of trials at each dot coherence level. The same random dot stimuli were repeated across the four conditions in a random order but new random stimuli were created for each session. There was a rest break in the middle of, and between, each block. Three subjects (1, 3, 4) completed six sessions for a total of 2004 trials in each of the four conditions. Subject 2 completed 2338 trials in each condition. Subject 2 was the first subject to experience the force-field condition. After initial analysis, the field strength was increased in order make the condition more effortful for the other subjects.

Prior to performing the main experiment the subjects were extensively trained (17–20 sessions with approximately 1000 trials per session) over a number of weeks to ensure they had stable performance in the components of the task. In general, it takes extensive practice for subjects to achieve a stable speed-accuracy regime. For this study we needed to compare the initial choices and reaction times across the effort conditions. We therefore chose to introduce those manipulations only after subjects had extensive practice. The training proceeded in several stages. First, subjects were trained to discriminate motion direction without a reaching movement. Fixed-duration (500 ms) stimuli were used to train subjects to discriminate dot motion direction and respond with a button press. Subjects' performance was assessed by logistic regression of probability of a rightward choice versus coherence extracting the sensitivity (slope) and bias. When both parameters were stable (5–8 sessions), subjects progressed to the next stage. Subjects then performed reaction-time training and were instructed to respond with a button press as soon as they reached a decision about the direction of dot motion. Subjects were assessed by their mean reaction times at the various coherence levels. When mean reaction times and accuracy remained stable (4–6 sessions), subjects progressed to the next stage. Next, subjects learned to make reaching movements of the appropriate duration without random dot stimuli. The subject initiated a trial by holding the cursor in the home position, and after a random delay, one of two choice targets turned green. If the subject moved to the target faster than 400 ms or slower than 600 ms, they received the error message, “Move slower” or “Move faster,” respectively. The three targets configurations were used as in the final experiment. Subjects progressed to the next stage if they performed fewer than 5 trials outside of the range of 400–600 ms in the last 30 trials. All subjects met these criteria after a single session and progressed to the main experiment. In the main experiment subjects performed reaction-time trials in which choice reach targets were visible from the onset of the trial.

### Data Analysis

**Figure 3:**
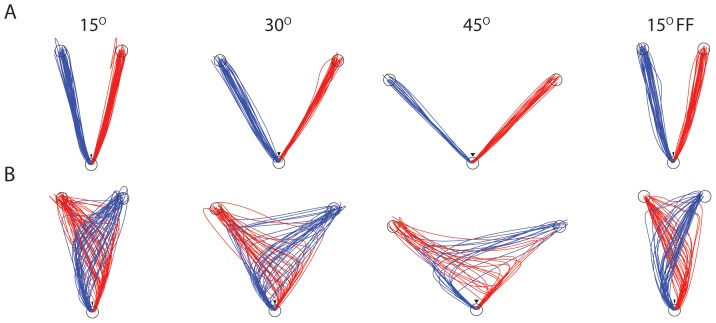
A: Sample hand trajectories from Subject 2 for the four conditions (3 different angular target separations and force field FF). Most trajectories extend directly from the home position (bottom circle) to one of the choice targets. B: In a fraction of trials, the trajectories change course during the movement, indicating a change of mind. Panel B shows all the change of mind trials for this subject and in panel A and equal number of non change-of-mind trials have been randomly selected. Note the visual/haptic wedge just above the home position which subjects were not allowed to contact.

We recorded the hand trajectories at 1,000 Hz. For each trial, we recorded the choice target and reaction time (RT; time to movement initiation from start of motion stimulus). In addition, we developed a measure, based on the hand trajectories, of whether subjects had changed their decision during the movement. Normally, hand movements for easy trials (high coherence) were straight to the target (Fig. 3A). A change of mind was reflected in a trajectory that passed the wedge on one side but ended at the choice target on the other side.

In addition to the model based analysis described below we pursued several model-free analyses. To test whether subjects' sensitivity to motion (for their initial choices) varied by condition, for each subject we compared each condition to the 15° condition by fitting a logistic model:
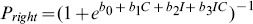
where C is the signed coherence, I is an indicator variable (0 for the 15° condition and 1 for the other test condition) and b_i_ are fitted coefficients. We evaluated the null hypothesis {Ho: b_3_ = 0} for 12 comparisons (4 subjects, 3 conditions) against a conservative level p = 0.01.

We performed a similar analysis to test whether changes of mind altered performance. To do this we examined whether the slope of the psychometric curve linking choice with signed coherence changed between initial and final decisions across the 4 conditions. We fit the equation above across all 4 conditions but now with the indicator variable, *I*, being zero for trials without a change of mind and 1 for trials with a change of mind. To test for a change in sensitivity (accuracy) with changes of mind, we evaluated the null hypothesis {Ho: b_3_ = 0}.

To examine whether reaction times varied across conditions, for each subject we compared the mean reaction time for each signed coherence in the 15° condition with the mean reaction times for each of the other three conditions (12 two-way ANOVAs with a conservative level p = 0.01).

To test whether changes of mind decreased with increasing effort we performed logistic regression of the probability of change of mind as a function of angular separation α for the three non-force field conditions:
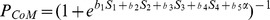
where all subjects were constrained to share the same linear effect of angle but could have different intercepts as determined by the indicator variable Si which was 1 for subject i and zero otherwise. We evaluated the null hypothesis {Ho: b5 = 0}. In addition we fit a fully saturated model, allowing for different effects of α for each subject. We performed a similar analysis to examine if the frequency of change of mind varied between the 15° and the 15° with force field conditions.

### Modeling

We first used the drift-diffusion model [15] to explain the proportion of initial choices and reaction times and then fit change-of-mind behavior using methods similar to [3].

For the initial choices, the model posits that evidence accumulates from a starting point, y_0_, until it reaches an upper or lower bound (±B), which determines the initial choice and decision time. The increments of evidence are idealized as Normally distributed random variables with unit variance per second and mean μ = kC+μ_0_, where C is signed motion strength (specified as the proportion of dots moving in net motion direction, positive = rightward and negative = leftward motion); k, B, y_0_ and μ_0_ are free parameters. The parameters B and k explain the tradeoff between speed and accuracy of the initial choices; μ_0_ and y_0_ are drift and starting point offsets, which explain bias (if any) for one of the choices. The reaction time incorporates additional latencies from stimulus onset to the beginning of the bounded accumulation process and from the termination of the process to the beginning of the motor response. The sum of these latencies, termed non-decision time, is modeled as a Normally distributed variable with mean t_nd_ and standard deviation σ_tnd_, truncated and renormalized so as to take on only positive values

Data were fit by maximizing the log likelihood of the responses (that is the probability of the initial choice at the reaction time, given the stimulus direction, coherence and model parameters). To achieve this for each trial we computed the probability of hitting the choice bound corresponding to the subject' choices for the possible decision times [6]. To calculate the likelihood of this choice at the particular reaction time observed on the trial we convolved this decision time distribution with the distribution of nondecision times (with mean t_nd_ and standard deviation σ_tnd_). This procedure calculates the summed probability of all possible combinations of possible nondecision times and decision times that would give the reaction time seen on that trial. This procedure was performed for each trial to calculate the log likelihood across the trials and we chose model parameter to maximize the log likelihood.

As there were minimal differences in the initial choice across conditions within a subject, we fit each subject's data using a model in which the 6 parameters were shared across the 4 conditions (k, B, y_0_, μ_0_, t_nd_, σ_tnd_). We performed multiple optimizations from over 50 different starting points to maximize the chances of the global optimum. We then performed bootstrap analysis (over 500 samples) to estimate the confidence limits on the fitted parameters.

#### Modeling change-of-mind

Because the stimulus duration in each trial equals the reaction time, there is additional evidence from the stimulus that is potentially available for processing after the brain has committed to an initial choice. We used a simple model (Fig. 2) that incorporates this additional information as follows. When the initial decision ends, the accumulation continues until either a second, post-initiation change-of-mind bound is crossed (offset by *B_com_* from the zero evidence in the opposite direction to the initial choice bound), in which case the decision is reversed, or a temporal deadline is exceeded (*t_pip_*) in which case the initial decision is reaffirmed.

The values of k and μ_0_ derived from the first model for initial choice were used with the drift equation to predict for each trial if a change of mind occurred and whether it produced a correct or incorrect final choice. The maximum-likelihood method used to determine the initial choice parameters was also used to estimate values of *B_com_* and *t_pip_* for a condition.

To examine the changes in *B_com_* and *t_pip_*, 95% confidence ellipses for *B_com_* and *t_pip_* were calculated from the data using the profile likelihood method (Appendix A in [16]). That is, the 1-α confidence ellipse encloses all values of *B_com_* and *t_pip_* for which the log likelihood is within 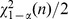 of the maximum log likelihood, where n is the number of parameters (2) being estimated via the method of maximum likelihood.

## Results

Four naive subjects were trained to decide the direction of motion in a dynamic random dot display and to indicate their decisions by moving a robotic handle to a left or right choice target (Fig. 1A). They controlled the viewing duration, effectively terminating the display by moving the handle outside the 0.5 cm radius home position. Since the initial movement was required to pass to the left or right of a virtual wedge, it was straightforward to classify the initial decisions as left or right. Whereas most trajectories continued to the corresponding choice target (Fig. 3A), a minority switched direction to the opposite choice target (Fig. 3B). These trials were designated change of mind trials (CoM).

**Figure 4:**
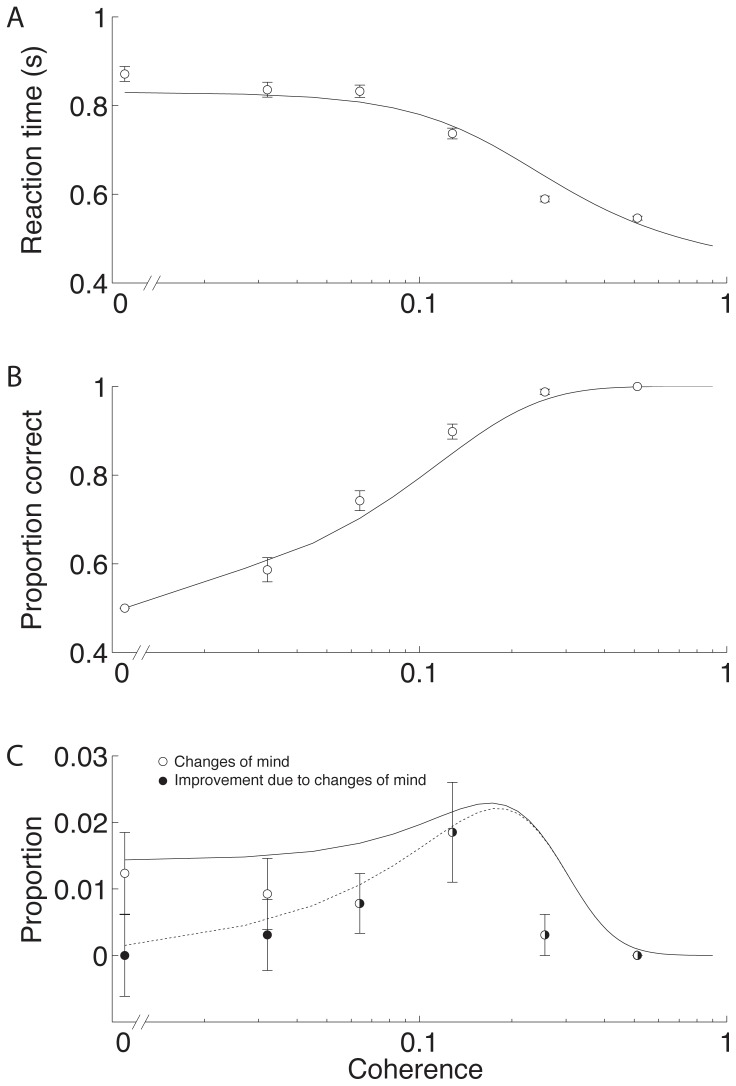
Psychometrics of choice for all subjects for the 30° condition. A: Reaction time as a function of motion coherence (specified as the proportion of dots moving in the same direction). Open circles are mean reaction times and solid lines are fits of the data to the drift-diffusion model. B: Proportion of correct trials as a function of motion coherence. The solid lines are the fits of the data. C: Proportion of trials with changes of mind as a function of motion coherence. Solid circles are for all changes of mind and open circles are the proportion of trials on which a change of mind improved performance (that is the difference in the number of changes of mind that correct an error and the number that induce an error). The solid and dotted lines are the fits of the extension of the drift-diffusion model for changes of mind to the data. Error bars show ±one standard error and are derived from the binomial distribution for the proportions.

Stronger motion induced faster reaction times (Fig. 4A) and more accurate choices (Fig. 4B), and as previously shown, both the accuracy of these initial choices and the reaction times were explained by a bounded evidence accumulation model (Fig. 4A & B, solid curves) [1]–[3]. Although the random dot motion disappeared at the moment of movement initiation, there were occasional changes of mind (Figure 4C solid circles show proportion of trials with a change of mind). Changes of mind tended to be most common at the low motion strengths, where initial errors were more frequent. Moreover, changes of mind were not random, but tended to improve accuracy (Fig. 4C open circles show proportion improvement due to change of mind).

These change-of-mind events can be explained by the additional processing on the stream of evidence that did not have time to influence the mechanism which mediated the initial choice. They are interesting because they expose a level of sophistication to the decision process that trades speed versus accuracy. The phenomenon suggests that the brain might control the speed-accuracy tradeoff differently when changes of mind are possible and not too costly. To test this, we introduced variations in the cost of CoM. We did this by changing the angular separation between choice target positions or by introducing a force field to impede crossing over from an initial left or right choice to the opposite target. We first show that these manipulations affected the frequency of change of mind and then attempt to determine whether these manipulations affect the processes underlying the initial decision, the revision after initiation, or both.

**Figure 5:**
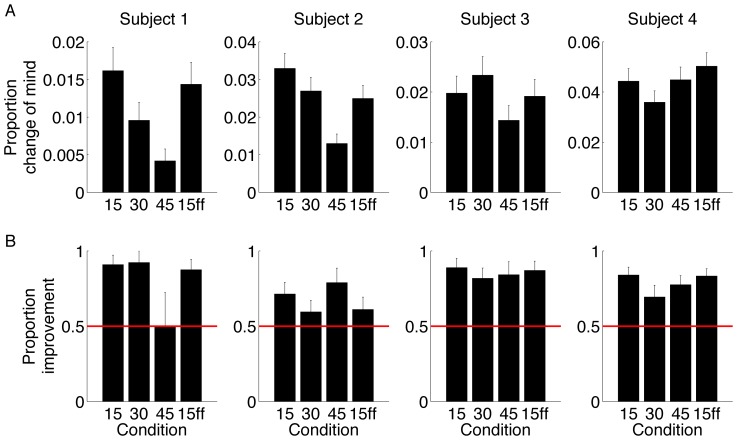
Changes of minds. A. Proportion of trials with a change of mind as a function of the condition for each subject. B: Proportion improvement on change of mind trials - that is the ratio of change of mind trials that correct an error to the total number of change of mind trials. Values exceeding one half (red bar) are consistent with an overall tendency to correct an initial error. Error bars are s.e.

Figure 5A tallies the proportion of CoM trials associated with each of the testing conditions. For three of the four participants, CoM were least common when the choice targets were separated by 45° To examine whether there was a reduction in changes of mind with effort we analyzed the three non force-field conditions in which we could order the effort from least (15°) to most effort (45°). We performed a logistic regression of the probability of change of mind across all 4 subjects allowing subject-dependent intercepts but requiring all subjects to share a common dependency on the angular target separation (see Methods for details). This showed that across the subjects there was a significant (p<0.0001) reduction in changes of mind with angular separation. Examining each subject individually showed that Subjects 1-3 each showed a significant reduction in changes of mind with angular separation. (P<0.001 for subjects 1 & 2, p = 0.02 for subject 3; subject 4 n.s.). We performed a similar analysis to examine whether the number of changes of mind varied between the 15° and the 15° with force field conditions—the results showed that there was no significant change in frequency.

For all subjects and conditions (bar 1), CoM led to an improvement in accuracy (Fig. 5B). The one exception is S1 whose rare CoM were consistent with chance performance in the 45° condition. We examined whether changes of mind improved the performance as reflected in the slope of the psychometric function for each subject (across the 4 conditions - see Methods). Changes of mind reliably improved accuracy for three of the subjects (Subjects 1, 3 & 4; p = 0.04, p<0.001, p<0.001 respectively; Subject 2 p = 0.08) by improving sensitivity to motion.

**Figure 6:**
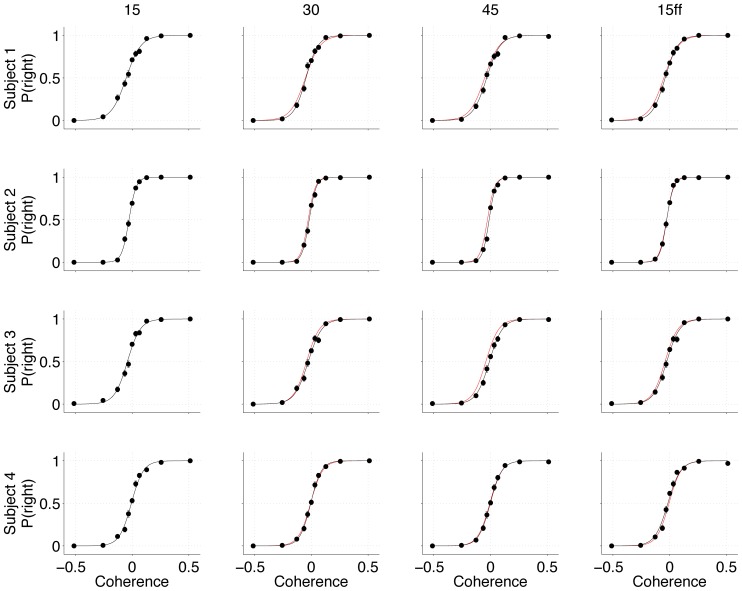
Initial choice function. Graphs show the probability of a rightward choice as a function of motion strength (sign of coherence reflects dot motion direction: left or right) for each subject and condition. Curves are logistic fits. For comparison the red curve for each subject is the logistic fit to their 15° condition. Error bars are s.e.

In broad terms, there are two ways that energetic costs of CoM could affect the decision process. The first is to induce a more conservative strategy behind the initial choice. For example, participants might have adopted a higher initial bound, leading to slower but more accurate initial choices or they might have increased attention to improve the accuracy of initial choices. Figure 6 shows the initial choice functions for all the conditions for the four subjects, fit with logistic regression. The graphs are displayed to facilitate comparison with the 15° condition (red curve). An increase in sensitivity would be apparent as a steeper slope of the psychometric function, and this was seen in just one case (Subject 1, 30° condition; p<0.001, uncorrected, see Methods for details). Note that in the drift-diffusion model, accuracy (hence sensitivity) is governed by the product of the bound height and the signal to noise (i.e., the k term that sets the drift term in the diffusion model). We also failed to observe systematic changes in the RT for the initial choice. In only two cases (Subject 1, 45° condition, and Subject 4, 15FF condition) did we observe evidence of slowed initial choices in the face of higher energy costs for CoM (ANOVA, main effect of condition, p<0.01), although the slowing was marginal (mean reaction time differences of 34 and 36 ms respectively). This implies that the bound height (B term in the drift diffusion model) was stable. We conclude from these analyses that the cost manipulations we imposed failed to affect the strategy subjects employed to make their initial decisions. As mentioned below, we do not believe this conclusion would hold generally, but it greatly simplifies the next analyses.

The second way for energetic costs to affect the frequency of CoM is to alter the processing of motion information in the post-initiation period. The simplest explanation of CoM is that the initial choice ignores a portion of the stream of evidence because commitment —which is informed by stimulus evidence a visual latency ago— occurs before the movement, which in turn terminates the display. The total non-decision time amounts to ∼400 ms worth of information, which can be used to revise the initial choice. A variety of potential mechanisms could exploit this additional information, but the simplest is continued accumulation of evidence after from the initiation bound to a new “change of mind” bound (*B_com_* Fig 2). The initial choice is reaffirmed if the accumulated evidence does not reach this CoM bound before the stream of evidence runs out or before an even earlier deadline (*t_pip_*). This is the model that generated the fits in Figure 4c (see also [3]).

**Figure 7:**
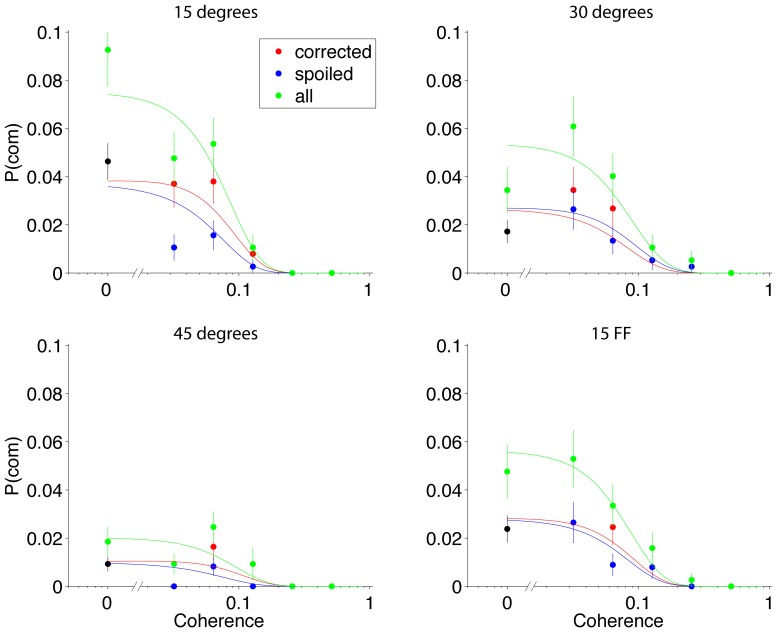
Pattern of changes of mind for Subject 2. Probability of a change of mind as a function of (unsigned) motion coherence for the four conditions. Green circles are all change of mind trials, red are initial errors that were corrected and blue are trials that spoiled an initially correct choice. Solid curves are model fits to the data for the change of mind model. Error bars are s.e.

We next examined whether *B_com_* and *t_pip_* change with the four effort conditions. For each subject, we adopted the parameters used to explain the initial choices (Table 1) and attempted to explain the fraction of CoM trials at each motion strength with the two degrees of freedom (*B_com_* and *t_pip_*). Figure 7 shows representative fits to the data from one subject. The fits clearly capture the frequency and pattern of CoM as a function of motion strength, and they approximate the breakdown of CoM by the correction of initially erroneous choices or spoiler of initially correct choices.

**Table 1:** Fitted parameters of the accumulation-to-bound model to the initial decisions.

	Subject 1	Subject 2	Subject 3	Subject 4
k	11.11 (10.42∶11.88)	21.93 (20.63∶23.14)	19.61 (18.61∶20.73)	19.55 (17.91∶21.39)
B	0.659 (0.650∶0.666)	0.827 (0.817∶0.836)	0.465 (0.458∶0.472)	0.467 (0.458∶0.476)
t_nd_	0.417 (0.414∶0.421)	0.481 (0.475∶0.487)	0.437 (0.433∶0.441)	0.483 (0.477∶0.489)
σ_tnd_	0.023 (0.020∶0.026)	0.061 (0.054∶0.067)	0.038 (0.035∶0.042)	0.059 (0.054∶0.063)
μ_0_	0.030 (0.025∶0.035)	0.024 (0.022∶0.027)	0.018 (0.013∶0.023)	0.014 (0.008∶0.019)
y_0_	0.097 (0.081∶0.112)	−0.083 (−0.101∶−0.065)	0.055 (0.031∶0.076)	−0.034 (−0.062∶−0.007)

The six parameters for initiation are shown; parentheses show the 95% confidence intervals obtained by bootstrapping.

**Figure 8:**
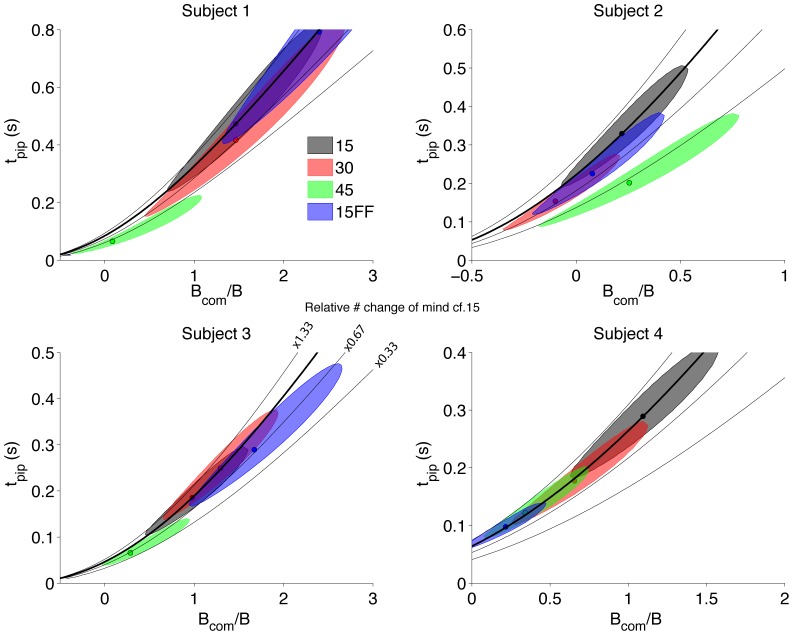
Parameter fits to the change of mind model for each subject. The change of mind model fits the change of mind bound B_com_ and the time allowed for post-initiation processing t_pip_. For each condition, the maximum likelihood estimates (small solid circles) are shown, as well as the 95% confidence region for the parameter estimate (shaded region). The proportion of change-of-mind isocontour lines are shown for the proportion of change of mind trials in the 15° target separation condition (solid line) as well as for proportion of change of mind trials that are 33, 67 and 133% that of the 15° separation condition.

Figure 8 summarizes the fits for all four subjects for all conditions by illustrating the 95% confidence regions for *B_com_* and *t_pip_* for each condition (colored regions). It is important to note that the same total number of CoM can be achieved by combinations of these parameters (contours, Fig. 8). For example, as post initiation time increases, it may be compensated by an increase in the distance to the CoM bound (*B_com_*). Thus, the confidence regions for the parameters tend to follow these iso-CoM contours. Importantly, we are able to fit *B_com_* and *t_pip_* uniquely (solid small circles Fig 8), because values that support the same total frequency of change of mind produce different patterns of changes of mind across the coherence levels. For 3 of the 4 subjects, the 45° condition stands out as distinct from the other conditions, consistent with the reduced frequency of CoM under this condition (Fig. 5a). That is, subjects required less evidence to change their mind in this condition (smaller *B_com_*) but integrate the information for a shorter time (reduced *t_pip_*) with this combination leading to a reduction in CoM. Interestingly, Subject 4, who did not change his frequency of CoM in a systematic way, appears to have changed strategy (that is their *B_com_* and *t_pip_*) to achieve this very consistency. Also as expected, for 3 of the 4 subjects, the fitted *t_pip_* is shorter than the fitted non-decison time (sum of sensory and motor delays) from the initial choices (437–483 ms) suggesting these subjects do not use all the information available in the post-initiation period. In contrast, Subject 1 has conditions (15° and 15FF) in which it appears he uses the entire information available (values of *t_pip_* much larger than the fitted non-decision time for this subject are probably spurious as reflected by the large confidence region which encompass reasonable values of *t_pip_*). Thus, for all subjects, we adduce that effort cost affected the post-initiation processing.

## Discussion

The random dot motion task is exemplary of a variety of decisions that benefit from an accumulation of evidence in time. It is well known that the criterion for terminating a decision instantiates the tradeoff between speed and accuracy [7], [8], [17]. When there is a steady stream of evidence, termination implies that the decision-maker must ignore a portion of the evidence stream when making her decision. This is because of the latency for new information to be incorporated into a decision variable and the latency from decision termination to the cessation of the evidence stream, which is at movement initiation in our experiment. The sum of these latencies, termed the non-decision time (t_nd_), was ∼400 ms in our experiments, consistent with previous studies in humans and monkeys [3], [7]. This value accords with neural recordings from the parietal cortex of the monkey, which exhibit an approximately 200 ms latency to the start of evidence accumulation [12] and latency from the signature of decision termination to the initiation of the behavioral response (∼70 ms for saccades [14], [18] and ∼170 ms for reaches [19]). Recently, it was shown that this ∼300–400 ms of ignored evidence is not lost entirely, but can be exploited to revise an initial choice [3]. Such changes of mind tend to improve accuracy because they are more likely to correct an initially erroneous choice than they are to spoil an initially correct choice. The present study shows that post-initiation processing is affected by the effort associated with a change of mind.

Subjects indicated their decisions by moving their hand from a central position to one of two choice targets. We designed the experiment to ensure that both the initial choice and a change of mind could be unambiguously discerned from the hand trajectories (Fig. 3). We manipulated the cost of CoM by controlling the angular separation of the choice targets or by introducing a viscous force field (FF) in the direction that would counter a CoM. Three of the four subjects reduced the frequency of change of mind trials when the angular separation between targets was greater, and all four changed their strategy when processing information after initiating the response. The absence of influence of FF might speak to the complexity of the effort cost (e.g., factors beyond force alone, such as trajectory, speed), but it might simply reflect the limited strength of the viscous field we employed.

Although energetic costs reduced the frequency of CoM by up to 90%, the overall effect was small, because subjects only changed their minds on 1–5% of trials on the least costly condition. We observed a greater number of CoM in an earlier study, likely because these subjects adopted a different speed accuracy tradeoff. In the present study, we specifically avoided encouraging subjects to adopt a faster and less accurate speed accuracy tradeoff at baseline, because we worried that it could give rise to fewer CoM under an energy cost simply by relaxing this regime to produce slower and more accurate initial choices. Although the price of our experimental strategy is fewer CoM, overall, the dividend is a clear interpretation. Energetic costs did not appear to affect the mechanism underlying the initial decision but instead altered processing of information after commitment to the initial choice, in the post-initiation period. By the same logic, there is no reason to believe that under different conditions, subjects would not adjust their initial decision strategy to compensate for post-initiation costs. For example, under speed stress, which tends to produce more CoM, we would expect energetic costs to slow initial choices. It remains to be seen if such a manipulation would have exposed an effect of the force field manipulation.

The small number of CoM in our study precludes a systematic comparison of alternative models to the one we applied. For example, in the brain there are at least two decision variables represented by populations of neurons that support the right and left choice, respectively. Hence, diffusion between two bounds is replaced with a race between two diffusion mechanisms [2], [6]. This implies that processing in the post-initiation period may not begin at the termination bound for the initial choice, but at a more intermediate value achieved by the losing mechanism. This idea invites further generalization once one entertains that possibility of multiple decision-making mechanisms operating in parallel.

Even in the restricted model framework we pursued, we cannot discern whether the reduction in change of mind was due to a change in their deadlines, the use of a more conservative bound for change of mind, or both. As shown by the contour lines in Figure 8, these two strategies can be traded against each other to achieve the same frequency of CoM. However, it is intriguing that the one subject who maintained a consistent CoM frequency in all conditions, did appear to change strategy to do so.

Decisions about actions naturally incorporate energetic costs. Minimizing these costs provides a rich framework for choosing among possible trajectories and online control of movement [20], [21]. Similarly, the potential values and costs associated with commitment to different propositions, depending perhaps on whether the decision was correct or not, may lead a rational decision-maker to bias her choices. This arises in medicine and finance, and it is captured in standard signal detection theory by a shift in decision criterion (e.g., accepting a high rate of false positive mammograms to avoid missing the rare cancer). From this perspective, it would not be at all surprising if differences in the energetic costs associated with communicating a decision were to introduce a choice bias. However, we chose to study a more subtle manipulation to see if energetic costs could impact a decision process without making either choice more desirable.

Since the advent of decision theory, and especially signal detection theory, it is common wisdom that decision criteria incorporate the value (or cost) of errors. The extension to sequential analysis incorporates deliberation time into the cost function [22], thereby inviting us to view the decision criterion as a policy on the tradeoff between speed and accuracy [11]. The present study extends this way of thinking into the post-initiation period, where energetic costs affect the criteria to revise an initial choice. Based on what we know of the underlying neurobiology, it is easy to accept that energetic costs associated with reporting a decision could affect the decision process. In monkeys, the decision variable is represented in structures that are associated with planning the motor response [14], [23] and the motor system of both human and nonhuman primates are affected by partial information leading to a decision [15], [19], [24]–[27]. In our study, the effect of effort was restricted to the epoch of processing after the initial decision. We predict that this will extend to the entire deliberation as this would be necessary to accommodate asymmetric energetic costs for left vs. right, but this remains to be seen.
